# Artificial Intelligence and Healthcare Policy: A Bibliometric Analysis of Global Research Trends

**DOI:** 10.3390/healthcare14142103

**Published:** 2026-07-14

**Authors:** Pegah Rashidian, Forough Heidarzad-Pahlaviani, Seyedsina Moghimnejadhosseini, Nikitha Chellapuram, Kavya Priya Somu, Saisree Reddy Adla Jala, Herby Jeanty, Satabdi Sahu, Abinash Mahapatro, Mahsa Talebzadeh, Mohammad-Javad Khosousi, Mohammad Amouzadeh-Lichahi, Ehsan Amini-Salehi, Ali Fatehi Hassanabad

**Affiliations:** 1 School of Medicine, Guilan University of Medical Sciences, Rasht 41448-95655, Iranmj.khosousi@gmail.com (M.-J.K.);; 2 Section of Digestive Diseases, Yale School of Medicine, New Haven, CT 06510, USA; 3 Faculty of Medicine, Semmelweis University, 1085 Budapest, Hungary; seyedsina.moghimnejadhosseini@stud.semmelweis.hu (S.M.); mahsa.talebzadeh@stud.semmelweis.hu (M.T.); 4 Cooper University Hospital, Cooper Plaza, Camden, NJ 08103, USA; 5 Centinela Hospital Medical Center, 555 E Hardy St, Inglewood, CA 90301, USA; 6 Mission Hospital, Asheville, NC 28801, USA; 7 The Brooklyn Hospital Center, Brooklyn, NY 11201, USA; 8 MKCG Medical College, Berhampur 760004, India; 9 SIU School of Medicine, Springfield, IL 62702, USA; 10 Division of Cardiac Surgery, Department of Surgery, Columbia University Irving Medical Center, New York, NY 10032, USA; 11 Section of Cardiac Surgery, Department of Cardiac Sciences, Libin Cardiovascular Institute, University of Calgary, Calgary, AB T2N 1N4, Canada

**Keywords:** artificial intelligence, health care policy, health policy, bibliometric analysis, science mapping, public health, machine learning, research trends, CiteSpace, VOSviewer, Biblioshiny

## Abstract

**Background:** Artificial intelligence is increasingly influencing health care and policy, yet the global research landscape linking artificial intelligence and health care policy remains underexplored. This study aimed to map publication trends, major contributors, collaboration networks, citation structures, and emerging themes in this field. **Methods:** A bibliometric analysis was conducted using the Web of Science Core Collection. The search was performed on 3 May 2026, and covered publications from 2000 to 3 May 2026. The final dataset included 347 peer-reviewed English-language original research and review articles. Biblioshiny, VOSviewer, and CiteSpace were used to analyze publication trends, country and institutional contributions, author and journal productivity, collaboration networks, citation and co-citation structures, keyword patterns, and thematic evolution. **Results:** Publications increased markedly after 2020 and reached their highest annual output in 2025. The 2026 publication count was lower because data for that year were partial at the time of database retrieval. Researchers from 82 countries and 900 institutions contributed to the field, with the United States leading in output, followed by China, England, Canada, and India. Harvard Medical School was the most productive institution, whereas Harvard University had the highest institutional centrality. *Frontiers in Public Health* published the most articles, and *PLOS ONE* was the most frequently co-cited journal. The most cited article was “Artificial intelligence and the future of global health.” Key research themes included machine learning, COVID-19, health policy, risk, large language models, interpretable machine learning, neural network-assisted screening, socioeconomic perspectives, and public health applications. **Conclusions:** Research on artificial intelligence and health care policy has expanded rapidly, particularly in recent years, and is increasingly centered on predictive modeling, public health decision-making, and emerging artificial intelligence technologies. These findings highlight influential contributors, evolving themes, and future directions for researchers, policymakers, and health care leaders.

## 1. Introduction

Over the past several decades, artificial intelligence (AI) has progressed from the development of computational systems inspired by human cognition to a far-reaching innovation that is reshaping clinical practice, population health, and the governance of health care systems [1,2,3]. In health care, AI-enabled approaches are increasingly applied to diagnosis, risk stratification, disease surveillance, outbreak prediction, and policy planning, thereby linking algorithmic innovation with decisions that affect both individuals and populations [1,4]. This expanding role has made AI increasingly relevant not only to medical practice but also to health care policy, including the design, organization, governance, financing, implementation, and regulation of health systems [2,5].

In this study, health care policy is used as an umbrella term referring to the decisions, regulations, governance structures, financing mechanisms, implementation strategies, and ethical frameworks that shape how health technologies and services are evaluated, adopted, delivered, and monitored within health systems. In the context of artificial intelligence, this includes policy dimensions related to governance and accountability, regulatory oversight, data privacy and security, public health planning, health system organization, resource allocation, financing and reimbursement, implementation readiness, ethical and equity considerations, and clinical policy decisions concerning the integration of AI-enabled tools into care pathways.

The policy significance of AI is particularly evident in public health, where data-intensive technologies may support epidemiological monitoring, resource allocation, risk communication, and evidence-informed decision-making [2,4]. Because policy decisions often require the integration of large, heterogeneous, and rapidly changing streams of clinical, demographic, and population-level data, AI has the potential to enhance the timeliness and analytical depth of health system responses [1,4].

However, the policy value of AI cannot be judged solely by predictive performance because its adoption also raises questions about accountability, transparency, equity, privacy, implementation capacity, and institutional readiness [2,4,5].

These concerns have prompted the development of formal ethical and governance frameworks for AI in health. The World Health Organization’s 2024 guidance emphasized that AI for health should be grounded in human rights, public benefit, transparency, responsibility, inclusiveness, and sustainability [2].

Similarly, the American Medical Informatics Association has argued that health AI governance should extend across the full lifecycle of AI systems, from design and implementation to monitoring, maintenance, and retirement [5].

Together, these frameworks indicate that the responsible integration of AI into health systems requires a shift from technology-centered evaluation toward governance-centered assessment.

The rapid emergence of generative AI and large language models (LLMs) has intensified the urgency of this policy debate [6].

Large multimodal and language-based models are increasingly being considered for clinical documentation, patient communication, medical education, workflow support, research assistance, and broader health system operations. At the same time, empirical evidence indicates that the evaluation of LLMs in health care remains fragmented, with limited use of real patient data and insufficient attention to fairness, bias, toxicity, and deployment readiness [7].

These limitations have strengthened calls for stronger validation standards, lifecycle monitoring, and regulatory oversight tailored to the distinctive risks of generative AI in medicine and public health.

The governance of health AI is also increasingly recognized as a global equity issue. Although AI may expand access, efficiency, and decision support in low- and middle-income settings, unequal regulatory capacity, uneven digital infrastructure, and asymmetries in data availability may deepen existing health disparities [8,9].

Accordingly, recent international initiatives have emphasized the need for collaborative, globally coordinated approaches to AI governance that promote safety, equity, implementation readiness, and context-sensitive policy development [9].

Against this backdrop, the scientific literature on AI in health care has expanded rapidly and become increasingly interdisciplinary. Several bibliometric studies have examined the broader development of AI in health care and medicine, showing sustained publication growth, increasing international collaboration, and the prominence of machine learning, deep learning, diagnostic applications, COVID-19-related research, and generative AI-related topics [10,11,12]. Other recent bibliometric analyses have focused on more specific domains, including AI and health information [13], AI in public health [14], responsible AI and digital health [15], AI governance [16], and AI and big data in health policy and management [17]. These studies have provided valuable insights into the technical, clinical, informational, ethical, and regulatory dimensions of AI-related health research.

However, most existing bibliometric studies have either addressed AI in health care broadly, focused on specific clinical or technical applications, or examined governance and public health as separate subdomains. Comparatively little attention has been given to the integrated research landscape at the intersection of AI and health care policy, particularly the combined analysis of publication trends, leading contributors, collaboration networks, citation and co-citation structures, thematic evolution, governance-related topics, public health planning, implementation, and health system policy dimensions. Therefore, a dedicated bibliometric analysis of AI and health care policy is needed to clarify how this interdisciplinary field has developed, which actors and sources have shaped it, and which emerging themes may guide future research and policy priorities [10,18].

Bibliometric analysis is well suited to address this gap because it enables systematic mapping of publication trends, influential contributors, collaboration networks, citation structures, and evolving research themes within a defined field. In biomedical research, such approaches are increasingly used to synthesize large bodies of literature, reveal intellectual structures, and identify emerging directions that may guide future scientific and policy priorities [19,20].

## 2. Aim and Specific Objectives of the Study

The aim of this study was to map and characterize the global research landscape at the intersection of AI and healthcare policy through a bibliometric analysis of publications indexed in the Web of Science Core Collection.

The specific objectives were to:

1. Quantify annual and cumulative publication trends in artificial intelligence and healthcare policy research;
2. Identify the leading countries, institutions, journals, and authors contributing to this field;
3. Examine international, institutional, and author-level collaboration networks;
4. Assess citation and co-citation structures to determine influential sources, authors, and publications; and
5. Identify major research themes, keyword patterns, thematic clusters, citation bursts, and emerging directions in the field.

By addressing these objectives, this study provides a structured overview of how research on AI and healthcare policy has evolved and highlights key contributors, knowledge structures, and future policy-relevant research directions.

## 3. Methods

### 3.1. Data Collection

Data on the included publications were retrieved from the Web of Science (WoS) Core Collection on 3 May 2026. This database was chosen because it provides extensive disciplinary coverage and is widely regarded as a reliable source for bibliometric analyses and systematic review-oriented investigations.

The search strategy was developed around two principal thematic domains: AI and health policy. To capture the AI dimension comprehensively, the search incorporated a wide range of related expressions, including “Artificial Intelligence,” “Machine Intelligence,” “Computational Intelligence,” “Computer Vision Systems,” “Knowledge Acquisition,” “Knowledge Representation,” “Machine Learning,” “Neural Networks,” and “prediction model.”

The health policy dimension was represented through terms such as “Health Policy,” “Health Policies,” “Health Care Policy,” “Healthcare Policy,” and “National Health Policy.” These two groups of keywords were systematically connected using Boolean operators to identify studies specifically addressing the intersection between AI-based technologies and health policy. The full search syntax is presented in Supplementary Table S1.

### 3.2. Eligibility Criteria and Study Selection

The eligibility criteria were defined before the screening procedure commenced. Publications were considered eligible only if they were peer-reviewed journal articles written in English and indexed in the WoS Core Collection. The dataset was restricted to original research articles and review articles that examined the use, relevance, influence, or broader consequences of AI within the context of health policy. Records that did not satisfy these conditions were excluded to preserve the methodological consistency and overall quality of the final dataset.

Several publication formats were deliberately omitted from the analysis. These included conference proceedings, editorials, letters, book chapters, meeting abstracts, preprints, news items, early access papers, and retracted publications. Such document types were excluded because they may differ substantially from full-length journal articles in terms of the rigor of peer review, completeness of reporting, and suitability for bibliometric assessment. Retracted papers were removed specifically to safeguard the credibility and validity of the evidence base. Articles published in languages other than English were also excluded because the bibliometric tools applied in this study are primarily optimized for English-language material, thereby supporting more uniform processing of keywords, citation relationships, and thematic structures.

The decision to retain only original articles and reviews was based on their generally greater methodological detail, stronger scholarly vetting, and more stable citation characteristics compared with other forms of academic output. Limiting the dataset to these two publication categories helped ensure that the final sample represented a robust and systematically indexed body of literature concerning AI and health policy.

The study selection procedure was performed independently by two reviewers and followed a structured screening process. After the initial application of the eligibility criteria related to document type and language, the remaining records were screened by title and abstract to determine their relevance to the study objective. Publications that did not address AI, health policy, or the intersection between these two domains were excluded. Any discrepancies between the two reviewers were discussed and resolved with input from a third reviewer. The screening process was conducted manually, without the use of automated screening tools.

Using the predefined search strategy, a total of 946 records were retrieved from the WoS Core Collection. In the initial refinement stage, 114 records were excluded because they did not satisfy the basic eligibility criteria. Among these, 100 were publication types other than original research articles or reviews, whereas 14 were written in languages other than English. Following this refinement, 832 records remained for further assessment.

**Figure 1:**
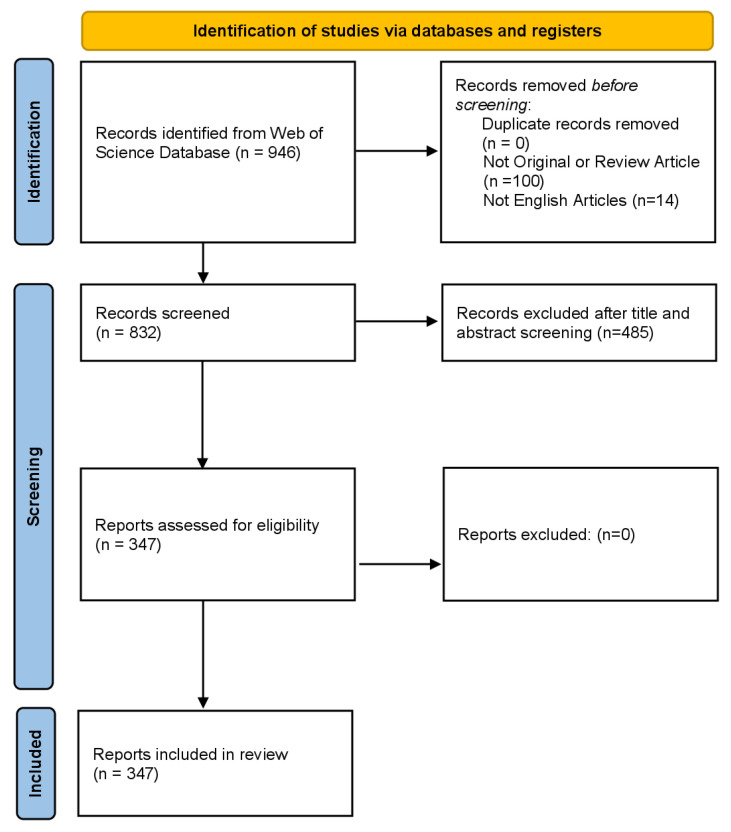
Study selection flow diagram. This figure illustrates the sequential procedure used to identify, screen, evaluate, and ultimately include eligible publications in the analysis. Each box represents a distinct stage of the selection process, and the connecting arrows show how records progressed through the workflow, indicating the main reasons for exclusion at each step.

These 832 records were then subjected to title and abstract screening to evaluate their relevance to the focus of the study. At this stage, 485 records were excluded because they did not sufficiently address the relationship between AI and health policy. After completion of the screening workflow, 347 publications fulfilled all inclusion criteria and were retained for the final bibliometric analysis, as illustrated in Figure 1.

### 3.3. Data Analysis

The bibliometric assessment was conducted using Biblioshiny, VOSviewer, and CiteSpace to analyze publications retrieved from the WoS Core Collection. After retrieval, the records were exported in both CSV and plain-text formats to ensure compatibility with the analytical platforms used in the study.

VOSviewer (Version 1.6.20 (0)) was employed to generate and visualize scientometric network maps. This software, developed by the Centre for Science and Technology Studies at Leiden University in the Netherlands, is commonly used to explore relational structures within academic literature. In the present analysis, VOSviewer was used to construct and visualize network-based relationships, particularly co-authorship and keyword-related network structures [21,22,23]. Association strength normalization was used to standardize link weights during the construction and visualization of bibliometric networks. Cluster detection was performed with a resolution value of 1.00 and a minimum cluster size of 1, and smaller clusters were combined automatically by the software. For layout generation, the advanced settings were configured with one random start and a maximum of 1000 iterations. Cluster optimization was carried out using 10 random starts and 10 iterations, whereas the remaining optimization parameters were kept at their default software settings.

CiteSpace (Version 7.0.R0 (64-bit)) was also used as a central tool for bibliometric mapping and visualization. As a Java-based application that integrates citation analysis with knowledge-domain visualization, CiteSpace is designed to uncover the intellectual structure and temporal progression of research fields. It supports the identification of highly influential studies, active research fronts, and newly emerging themes through citation-based network analysis. In this study, CiteSpace was especially valuable for examining citation dynamics and tracing the evolution of knowledge within the field over time.

A further contribution of CiteSpace was the calculation of betweenness centrality, which measures the extent to which a node functions as a connector between different parts of a network. Depending on the analysis, a node may correspond to an author, country, institution, journal, or publication. Nodes with higher centrality values are interpreted as occupying more strategic positions because they help link otherwise separate research communities, thematic areas, or citation clusters.

CiteSpace was also used for cluster analysis. Publications sharing similar co-citation characteristics were grouped into clusters to reveal the principal thematic domains represented in the literature. Cluster naming was carried out using the log-likelihood ratio approach, which extracts the most representative terms from the documents contained within each cluster. The reliability and distinctiveness of these clusters were assessed using modularity and silhouette indicators. Modularity describes the degree to which the overall network is divided into clearly separated subgroups, with values approaching 1 indicating stronger cluster separation. The silhouette coefficient evaluates the internal homogeneity of each group, with higher scores reflecting more coherent and well-defined clusters.

Temporal patterns were explored through CiteSpace’s time-slicing function, which partitions the dataset into predefined intervals and enables examination of how themes develop across different periods. CiteSpace was applied to perform thematic clustering, assess node centrality, and visualize the temporal development of the research field. The analysis covered the period from 2000 to 2026, with the data divided into one-year time slices. Terms were extracted from article titles, abstracts, author keywords, and Keywords Plus. Within each time slice, network connections were calculated using cosine similarity. Node selection was based on the g-index method with a scaling factor of k = 25. No pruning procedure was applied during network generation [24,25,26].

In the present study, CiteSpace was used for centrality-based network analysis, dual-map overlay visualization, temporal mapping, citation-burst detection, and cluster-based identification of emerging research fronts. 

To complement these analyses, Biblioshiny (Version 5.0), the web-based graphical interface of the Bibliometrix R package, was used as an interactive platform for descriptive and network-oriented bibliometric evaluation. It supported the generation of summary indicators, construction of bibliographic networks, and visualization of relational patterns in the dataset. Biblioshiny was particularly useful for examining collaboration networks, thematic structures, and research groupings. Its clustering and visualization capabilities, including approaches such as Louvain, Walktrap, and multidimensional scaling, assisted in identifying communities within bibliometric networks and clarifying relationships among topics, researchers, institutions, and countries [27]. Biblioshiny was used primarily for descriptive bibliometric analysis, source-level evaluation, country-level collaboration mapping, journal productivity assessment, co-citation summaries, and thematic visualization. 

## 4. Results

### 4.1. Publication Trend

**Figure 2:**
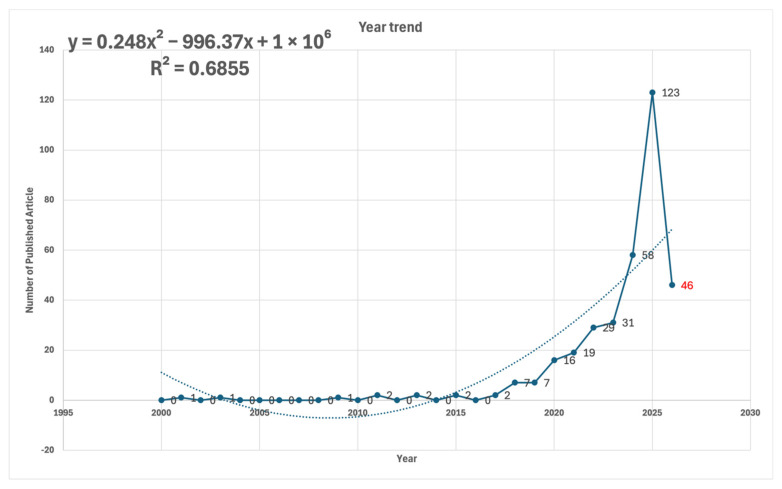
Annual publication output on AI and healthcare policy. This figure depicts the annual number of publications in the field from 2000 to 2026. The overall pattern indicates a gradual increase in research activity over time, followed by a particularly pronounced rise in publication volume during the most recent years.

The examination of publication patterns revealed pronounced overall growth in scholarly output throughout the study period. As illustrated in Figure 2, research activity remained minimal during the initial years, with annual production between 2000 and 2016 generally ranging from no publications to only a small number of articles. A more evident upward shift emerged after 2017. The number of publications rose from 7 articles in 2017 to 16 in 2020, marking the beginning of a more sustained expansion of the literature.

This growth became substantially more prominent from 2021 onward. The annual number of publications increased from 19 in 2021 to 29 in 2022 and 31 in 2023, before rising sharply to 58 articles in 2024. The greatest yearly output was recorded in 2025, when 123 publications were published, highlighting a marked acceleration of academic interest in this research area. In 2026, publication output declined to 46 articles. This reduction should be interpreted cautiously, as it may be attributable to incomplete database indexing or to the fact that the year was still ongoing when the data were retrieved. Despite this temporary decrease, the fitted polynomial curve continued to indicate an overall upward trajectory. The corresponding R^2^ value of 0.6855 suggests a moderate degree of fit between publication year and annual scientific output.

**Figure 3:**
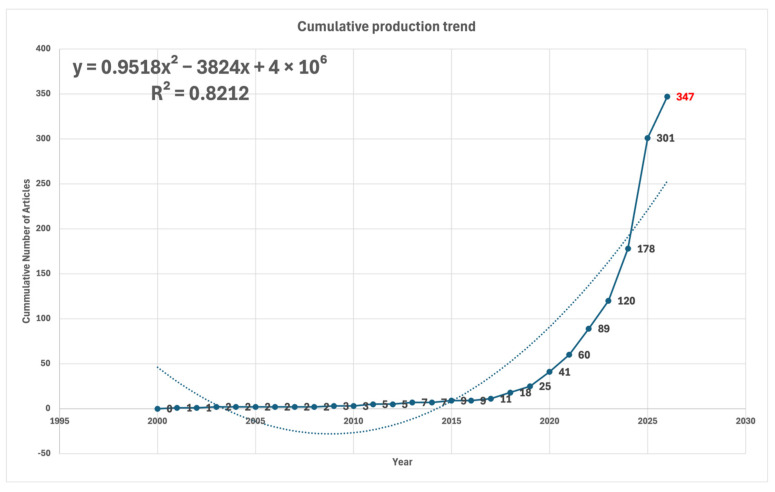
Cumulative growth of publications on AI and healthcare policy. This figure illustrates the cumulative number of peer-reviewed studies published between 2000 and 2026. The steadily ascending curve reflects sustained expansion in scientific output, with the rate of growth becoming notably more rapid in the most recent years of the study period.

The cumulative publication pattern, displayed in Figure 3, further emphasized the progressive and increasingly rapid development of the field. During the earlier stage, the total number of publications accumulated slowly, reaching only 9 studies by 2015. Thereafter, the pace of growth became more noticeable. The cumulative count increased to 25 publications in 2019, 41 in 2020, 60 in 2021, 89 in 2022, and 120 in 2023. This upward movement accelerated further in the following years, reaching 178 publications in 2024, 301 in 2025, and ultimately 347 publications in 2026.

Taken together, these results demonstrate a substantial expansion of research activity, particularly during the most recent phase of the study period. The cumulative growth curve displayed a consistently increasing pattern, with an R^2^ value of 0.8212, indicating a strong relationship between time and the accumulated volume of scientific production. Although year-to-year publication counts showed some variation, the cumulative evidence points to a persistent and intensifying scholarly focus on this topic.

### 4.2. Countries and Institutions

**Figure 4:**
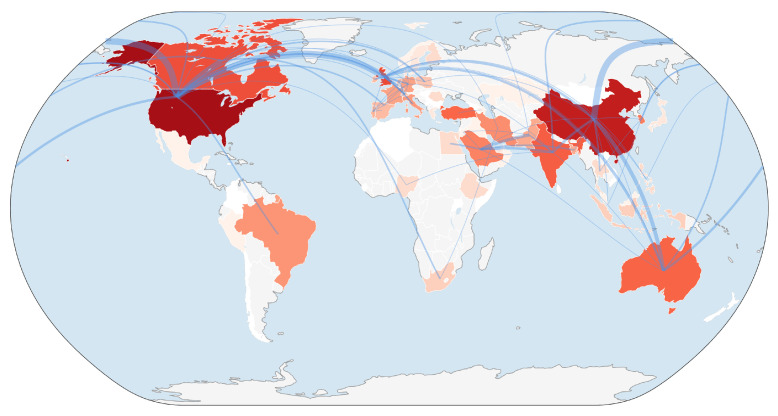
Worldwide country-level collaboration network in AI and healthcare policy research. Processed using Biblioshiny. This map shows the geographic distribution of countries contributing to the field and their international collaboration links. Country shading represents publication output, with darker red indicating a higher number of publications and lighter red indicating lower publication output. Blue curved lines represent international co-authorship links between countries, and thicker or more prominent lines indicate stronger collaborative relationships.

Research on AI and health policy involved contributions from 82 countries and 900 institutions. The international collaboration network at the country level is illustrated in Figure 4. In terms of publication volume, the United States emerged as the leading contributor, producing 102 documents. China followed with 58 documents, whereas England ranked third with 30 documents. Canada and India also demonstrated substantial output, contributing 28 and 24 documents, respectively.

Several additional countries played meaningful roles in the development of this literature. Australia produced 22 documents, followed by Türkiye with 20, South Korea with 16, Saudi Arabia with 15, and Iran with 13. Taken together, these findings show that research production in this area was dominated by the United States, China, and England, although a broader group of countries also contributed noticeably to the expansion of the field (Table 1).

To evaluate the structural importance of countries within the international collaboration network, centrality was examined. This indicator reflects the extent to which a country acts as a bridge linking different parts of the network. Countries with higher centrality scores are therefore interpreted as occupying more influential and connective positions in global research collaboration.

**Figure 5:**
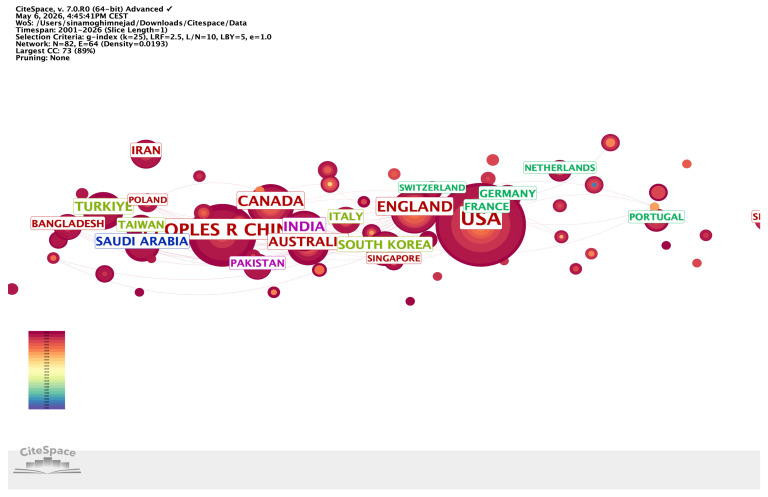
Country-level centrality network in AI and healthcare policy research. Processed using CiteSpace. This CiteSpace network visualizes the structural role of countries within the international collaboration network. Each node represents a country, and larger nodes indicate countries with greater prominence in the network. Connecting lines represent collaboration links between countries. The color gradient reflects the temporal distribution of collaboration activity across the analyzed period, with cooler colors representing earlier time slices and warmer colors representing more recent time slices. Because the automatically generated CiteSpace color legend is visually compact and centrality rings are not clearly distinguishable in the embedded figure, the exact betweenness centrality values should be interpreted using Table 2. In this figure, the main visual interpretation should focus on relative node size, country labels, and collaboration links.

According to Figure 5 and Table 2, the United States recorded the highest centrality value, at 0.43, indicating its dominant role not only in research productivity but also in connecting international collaborators. England held the second most central position, with a score of 0.30, followed by Australia, with 0.20. Saudi Arabia also displayed a notable bridging function, achieving a centrality value of 0.12, whereas China registered a score of 0.10.

Additional countries with meaningful centrality included Italy and Israel, both with values of 0.09, as well as India and Germany, each scoring 0.08. Türkiye and Bangladesh each recorded a centrality value of 0.07, whereas Canada had a score of 0.06. Overall, these results indicate that the United States, England, and Australia held the most strategically important positions within the international collaboration network. Their high centrality values suggest a strong capacity to connect otherwise separate national research communities and facilitate cross-border scholarly activity in the domain of AI and health policy.

The institutional analysis showed that Harvard Medical School was the most productive organization in research on AI and health policy, contributing 8 publications. Harvard University and the Harvard T.H. Chan School of Public Health ranked next, with 7 publications each. The University of Toronto, Duke University, and National Taiwan University each produced 6 publications.

A further group of institutions also made notable contributions. The National University of Singapore, Boston University, and the Ministry of Health each published 5 articles. In addition, Sun Yat-sen University, University College London, the University of New South Wales, the Massachusetts Institute of Technology, the University of Oxford, Johns Hopkins University, and Stanford University each contributed 4 publications. These findings indicate that influential universities and public health-oriented organizations, especially those located in North America and Asia, were major contributors to scholarly output in this domain (Table 1).

Institutional centrality was also assessed to determine the extent to which organizations served as connectors within the collaboration network. Harvard University recorded the highest centrality value, at 0.04, suggesting that it occupied the most prominent bridging position among institutions. The University of London followed with a centrality score of 0.02.

**Figure 6:**
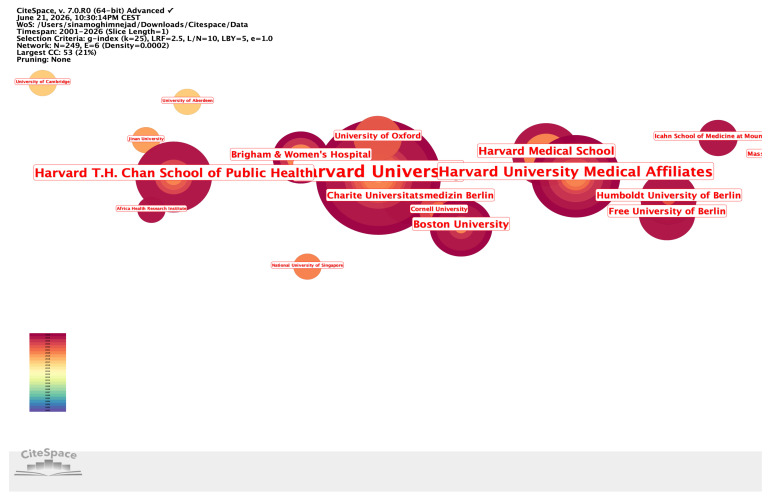
Institutional centrality network in AI and healthcare policy research. Processed using CiteSpace. This CiteSpace network visualizes the structural position of institutions within the collaboration network. Each node represents an institution, and larger nodes indicate institutions with greater prominence in the network. Connecting lines represent collaboration links between institutions. The color gradient reflects the temporal distribution of institutional collaboration activity across the analyzed period, with cooler colors representing earlier time slices and warmer colors representing more recent time slices. Because the automatically generated CiteSpace legend is visually compact and centrality rings are not clearly distinguishable in the embedded figure, the exact institutional betweenness centrality values should be interpreted using Table 2. In this figure, the main visual interpretation should focus on relative node size, institutional labels, and collaboration links.

Several other organizations demonstrated lower but still observable centrality values of 0.01. These included Harvard University Medical Affiliates, the Harvard T.H. Chan School of Public Health, Duke University, Boston University, Columbia University, the Australian National University, and Sun Yat-sen University. Overall, the results suggest that Harvard University held the most strategically central position in the institutional collaboration structure, whereas a broader group of universities also contributed to linking different research communities within the field of AI and health policy (Figure 6; Table 2).

### 4.3. Journals and Co-Cited Sources

**Figure 7:**
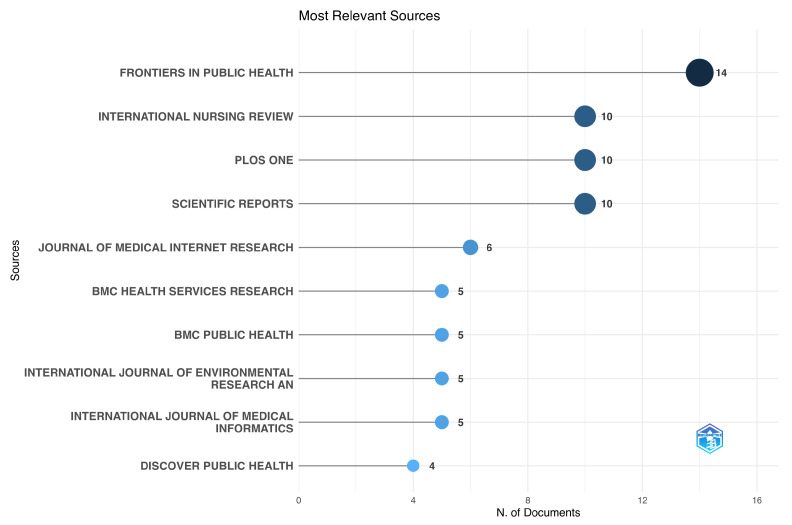
Top 10 journals by number of publications in AI and healthcare policy research. Processed using Biblioshiny.

The journal analysis identified 245 distinct sources that contributed publications to the literature on AI and health policy. As shown in Figure 7, *Frontiers in Public Health* was the most prolific journal, publishing 14 articles in this area. It was followed by *International Nursing Review*, *PLOS ONE*, and *Scientific Reports*, each of which contributed 10 publications. The *Journal of Medical Internet Research* ranked next, with 6 articles.

Several other journals also played a notable role in disseminating research on this topic. *BMC Health Services Research*, *BMC Public Health*, the *International Journal of Environmental Research and Public Health*, and the *International Journal of Medical Informatics* each published 5 articles. *Discover Public Health* was also among the more active outlets, contributing 4 publications.

**Figure 8:**
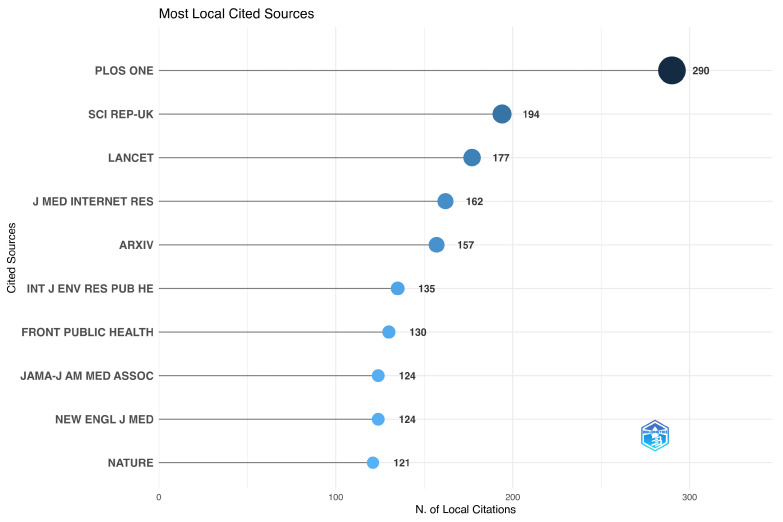
Top 10 co-cited journals in AI and healthcare policy research. Processed using Biblioshiny.

The co-citation analysis identified 8046 journals referenced across the dataset. As illustrated in Figure 8, *PLOS ONE* was the most frequently co-cited source, with 290 co-citations, highlighting its substantial influence within the examined literature. *Scientific Reports* ranked second, with 194 co-citations, followed by *The Lancet*, with 177.

**Figure 9:**
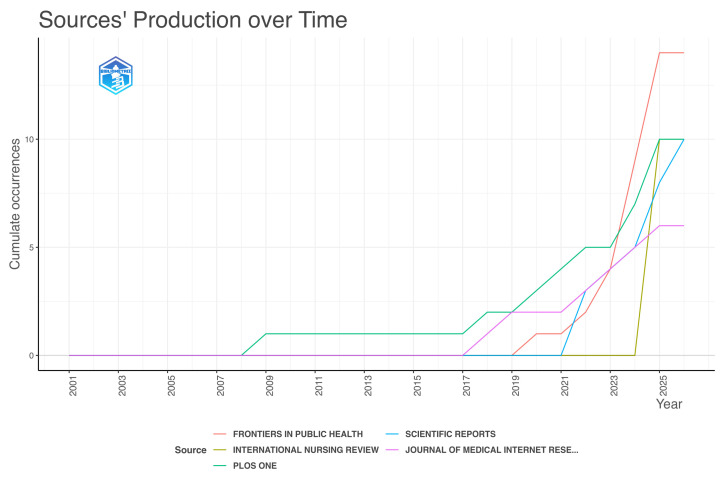
Journal production over time in AI and healthcare policy research. Processed using Biblioshiny.

The *Journal of Medical Internet Research* also occupied a prominent position, receiving 162 co-citations, whereas *arXiv* ranked fifth, with 157. Other highly co-cited sources included the *International Journal of Environmental Research and Public Health*, with 135 co-citations; *Frontiers in Public Health*, with 130; *JAMA*, with 124; *The New England Journal of Medicine*, with 124; and *Nature*, with 121. The temporal distribution of journal publications is presented in Figure 9.

**Figure 10:**
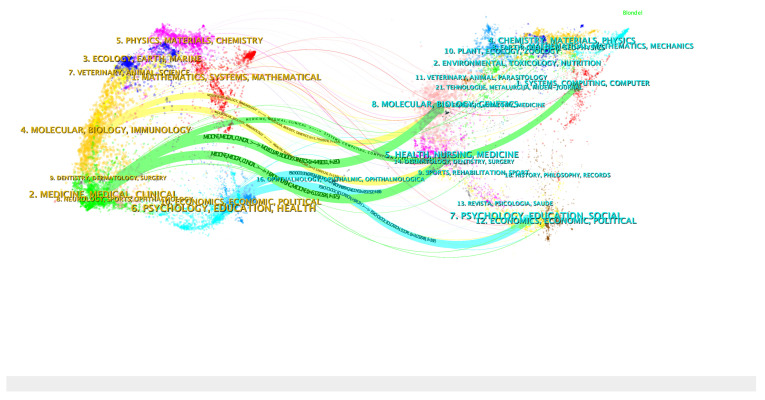
Dual-map overlay of citation paths in AI and healthcare policy research. Processed using CiteSpace. The left side shows the journal domains of the citing articles, and the right side shows the journal domains of the cited references. The main citation paths connect “Medicine, Medical, Clinical” and “Psychology, Education, Health” journals on the citing side with “Health, Nursing, Medicine” and “Psychology, Education, Social” journals on the cited side, indicating that the field draws mainly on medical, public health, clinical, nursing, psychological, educational, and social science literature.

A dual-map overlay was used to illustrate citation relationships between citing and cited journal domains. As shown in Figure 10, the left side of the map represents the journals in which the analyzed articles were published, whereas the right side represents the journals most frequently cited by those articles. The most prominent citation pathways originated from journals in the “Medicine, Medical, Clinical” and “Psychology, Education, Health” domains and extended mainly toward journals in the “Health, Nursing, Medicine” and “Psychology, Education, Social” domains. Additional citation links were observed between molecular biology, genetics, systems, computing, and related scientific domains. These patterns indicate that research on artificial intelligence and health care policy is grounded primarily in medical, public health, nursing, clinical, psychological, educational, and social science literature, while also drawing on computational and biological sciences.

### 4.4. Authors and Co-Cited Authors

**Figure 11:**
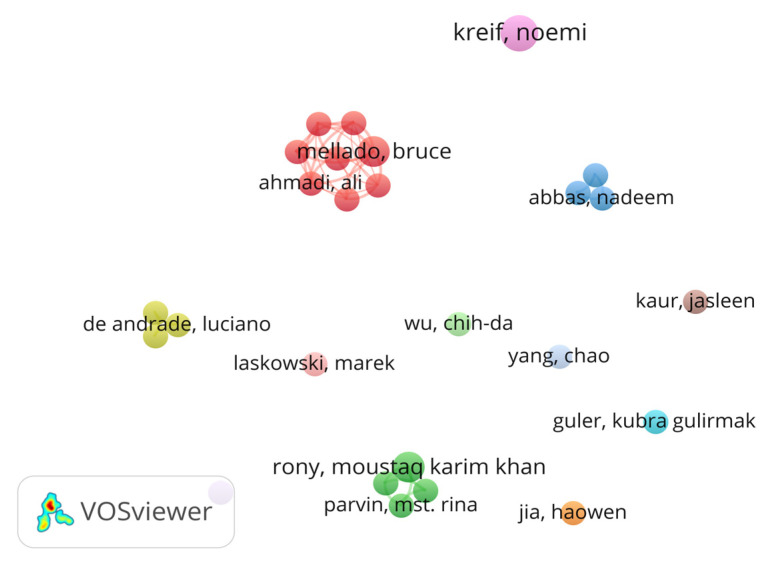
Co-authorship network of prominent authors in AI and healthcare policy research. Each node corresponds to an author contributing to the field. Node size represents publication productivity, whereas the thickness of connecting lines reflects the strength of co-authorship ties. Distinct colors identify collaboration clusters, highlighting groups of researchers who frequently publish together. Authors located closer to one another indicate stronger or more intensive collaborative relationships. Processed using VOSviewer.

The authorship analysis showed that 1733 authors contributed to the literature on AI and health policy. Among these contributors, Noemi Kreif was the most prolific author, with 4 publications. Bruce Mellado and Moustaq Karim Khan Rony followed, each producing 3 publications. A further group of authors contributed 2 publications each, including Luciano De Andrade, Oscar Kenji Nihei, Joao Ricardo Nickening Vissoci, Chih-Da Wu, Mitun Debnath, Mst. Rina Parvin, and Md. Wahiduzzaman. These researchers represented the most active authors within the analyzed dataset. The collaboration patterns among the leading authors are visualized in Figure 11 and summarized in Table 3.

Author citation analysis revealed that Moustaq Karim Khan Rony had the greatest citation impact, receiving 94 citations. Mitun Debnath, Mst. Rina Parvin, and Md. Wahiduzzaman followed closely, with 90 citations each. Marek Laskowski ranked next, with 47 citations, whereas Bruce Mellado and Chao Yang each accumulated 44 citations.

Several other authors also demonstrated considerable citation influence. Ali Ahmadi, Ali Asgary, Jude Kong, Blessing Ogbuokiri, and James Orbinski each received 36 citations. In addition, Nicola Luigi Bragazzi, Jianhong Wu, and Noemi Kreif each recorded 31 citations (Table 3).

The co-citation analysis identified 15,408 co-cited authors, reflecting the intellectual foundations of the field. L. Breiman was the most frequently co-cited author, with 46 citations. This was followed by S. Kumar, with 21 citations, and T. Q. Chen, with 20 citations. M. K. K. Rony, S. Athey, and S. M. Lundberg each received 19 citations, followed by Y. Liu, with 18 citations; Z. Obermeyer, with 17 citations; M. Kuhn, with 16 citations; and Y. Wang, with 13 citations. Collectively, these highly co-cited authors represent important intellectual influences in the field (Table 3).

### 4.5. Top Cited Articles

The citation analysis of key references in AI and health policy research revealed a group of highly influential publications. The most frequently cited work was “Artificial intelligence and the future of global health” by Schwalbe and Wahl (2020) [1], which accumulated 471 citations. The second-ranked paper was “Artificial intelligence versus clinicians in disease diagnosis: systematic review” by Shen et al. (2019) [28], with 231 citations.

The third most cited study was “Artificial intelligence framework for simulating clinical decision-making: a Markov decision process approach” by Bennett and Hauser (2013) [29], which received 210 citations. This was followed by “The Human Behaviour-Change Project: harnessing the power of AI and machine learning for evidence synthesis and interpretation” by Michie et al. (2017) [30], which was cited 160 times. Ranked fifth was “Forecasting the dynamics of cumulative COVID-19 cases for top-16 countries using statistical machine learning models” by Arunkumar et al. (2021) [31], with 157 citations.

Together, these highly cited studies reflect major intellectual contributions to the field, addressing themes such as global health transformation, AI-assisted diagnosis, clinical decision support, evidence synthesis, public health forecasting, and broader applications of machine learning in health care and policy-related contexts. The ten most highly cited papers are summarized in Table 4.

### 4.6. Keyword Trends

Keyword analysis is a central element of bibliometric studies, as it helps reveal the major research themes, conceptual priorities, and evolving directions within a given field. In the literature on AI and health policy, “machine learning” emerged as the most frequently used keyword, appearing 94 times. This was followed by “artificial intelligence,” which occurred 76 times. The term “COVID-19” ranked third, with 35 occurrences, underscoring the considerable influence of pandemic-related research on the development of this field.

**Figure 12:**
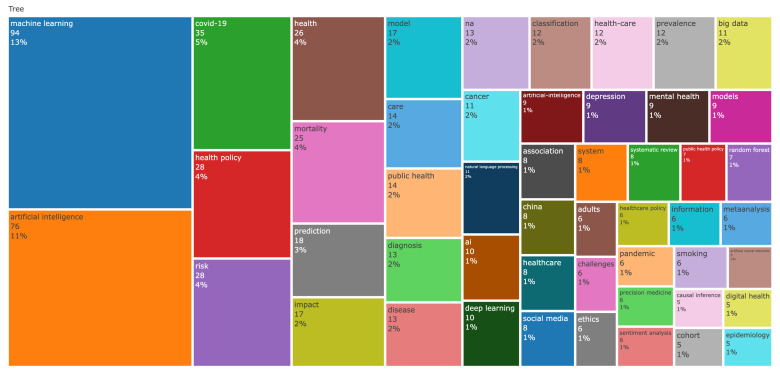
Treemap of the most frequent keywords in AI and healthcare policy research. This figure displays the keywords that appeared most frequently across the analyzed literature. The relative size of each rectangle corresponds to the frequency of occurrence, with larger areas representing terms used more extensively in the dataset. Processed using Biblioshiny.

Several other keywords also appeared frequently. “Health policy” and “risk” were each recorded 28 times, whereas “health” appeared 26 times and “mortality” occurred 25 times. The keyword “prediction” was identified 18 times, and both “impact” and “model” were observed 17 times. Additional terms of note included “care” and “public health,” each with 14 occurrences, as well as “diagnosis” and “disease,” which appeared 13 times each (Figure 12).

**Figure 13:**
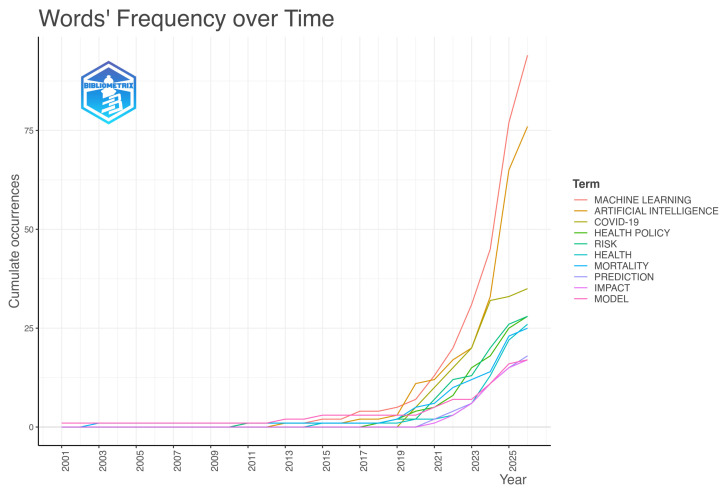
Keyword frequency over time in AI and healthcare policy research. Processed using Biblioshiny.

Figure 13 illustrates the cumulative frequency of the most frequently occurring keywords over time. The temporal pattern shows that keyword activity remained relatively low and stable during the early years of the study period, with limited cumulative growth before 2018. From approximately 2019 onward, keyword occurrence began to increase more noticeably, followed by a pronounced acceleration after 2021. “Machine learning” showed the steepest increase and became the term with the highest cumulative frequency by the end of the study period, indicating its central role in the development of research on artificial intelligence and health care policy. “Artificial intelligence” also demonstrated a strong upward trajectory, particularly after 2023, reflecting the growing prominence of AI-related terminology in the field.

“COVID-19” emerged as an important keyword after 2020 and showed rapid cumulative growth, suggesting that the pandemic contributed substantially to the expansion of AI-related research in health policy and public health contexts. Other terms, including “health policy,” “risk,” “health,” “mortality,” “prediction,” “impact,” and “model,” also increased over time, although at a more moderate pace. Overall, the figure indicates a shift from limited early keyword activity toward rapid thematic expansion in recent years, with machine learning, artificial intelligence, COVID-19, prediction, risk, and health policy representing major recurring themes within the literature.

**Figure 14:**
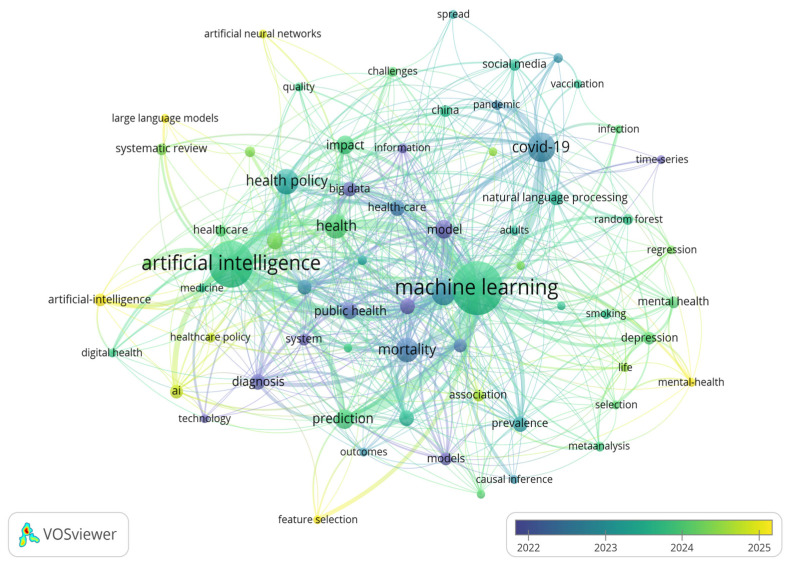
Temporal overlay map of keywords in AI and healthcare policy research. This figure depicts the evolution of keyword usage across the literature. Node size corresponds to the frequency with which each keyword appears, whereas line thickness represents the strength of co-occurrence between terms. The color gradient reflects the average publication year associated with each keyword, with cooler colors representing earlier topics and warmer colors representing more recent topics, thereby distinguishing established themes from emerging areas of interest. Keywords located closer to one another indicate stronger conceptual associations and more frequent joint occurrence. Processed using VOSviewer.

Figure 14 presents the temporal overlay map of keyword co-occurrence generated using VOSviewer. In this visualization, node size reflects the frequency of keyword occurrence, link thickness indicates the strength of co-occurrence relationships, and node color represents the average publication year associated with each keyword. Earlier keywords are shown in darker colors, whereas more recent keywords are shown in yellow.

The map shows that “machine learning” and “artificial intelligence” were the most prominent and highly connected keywords, indicating their central role in the research field. These terms were strongly linked to “health policy,” “public health,” “health,” “prediction,” “mortality,” “model,” “system,” and “COVID-19,” suggesting that the literature connects AI-based methods with public health decision-making, risk assessment, predictive modeling, and health care policy applications.

The temporal overlay also indicates a gradual shift in research focus over time. Early and mid-period themes were more strongly associated with terms such as “public health,” “model,” “mortality,” “system,” “prevalence,” and “COVID-19.” More recent keywords, shown in yellow-green to yellow tones, included “large language models,” “artificial neural networks,” “systematic review,” “AI,” “feature selection,” “mental health,” and “random forest.” This pattern suggests increasing attention to advanced AI techniques, generative AI-related topics, machine learning model development, and evidence synthesis in the most recent phase of the field.

**Figure 15:**
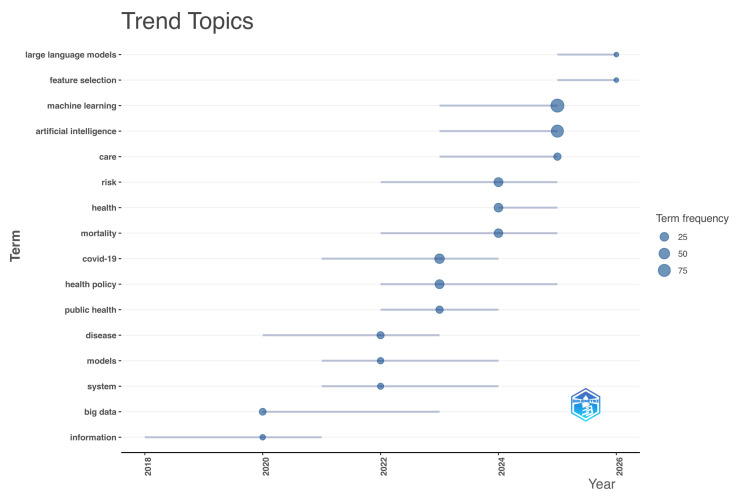
Keyword-based trend topics in AI and healthcare policy research. This figure shows how prominent research themes changed over time within the literature. Larger circles denote keywords with greater frequency, whereas horizontal lines indicate the time span during which each topic was particularly prominent in the field. Processed using Biblioshiny.

Figure 15 presents the trend topics identified across the study period. The figure shows the temporal distribution of frequently occurring terms, with the size of each circle indicating term frequency and the horizontal line representing the period during which each term appeared prominently in the literature. Earlier trend terms included “information” and “big data,” which appeared around 2020, suggesting early attention to data-driven approaches and information-related aspects of AI in health care policy research.

From 2022 onward, the thematic focus broadened to include “disease,” “models,” and “system,” followed by a stronger emphasis on “health policy,” “public health,” and “COVID-19” around 2023. These terms indicate increasing attention to the application of AI-related methods in health systems, public health decision-making, and pandemic-related policy contexts. By 2024, terms such as “risk,” “health,” and “mortality” had become more prominent, reflecting interest in predictive modeling, population health outcomes, and risk assessment.

The most recent trend topics, appearing mainly in 2025 and extending toward 2026, included “machine learning,” “artificial intelligence,” “care,” “feature selection,” and “large language models.” Among these, “machine learning” and “artificial intelligence” had the highest term frequencies, highlighting their dominant role in the field. The emergence of “large language models” and “feature selection” in the most recent period suggests growing interest in advanced AI methods, model optimization, and generative AI-related applications.

### 4.7. Cluster Analysis

Cluster analysis was performed using CiteSpace to identify the main thematic groupings within the literature on AI and health care policy. The cluster labels shown in the visualization were generated automatically by CiteSpace using the log-likelihood ratio approach and should therefore be interpreted as software-derived thematic indicators rather than manually assigned topic names. To improve interpretability, the labels were reviewed in relation to the surrounding keywords, connected nodes, and broader thematic context of the clusters.

**Figure 16:**
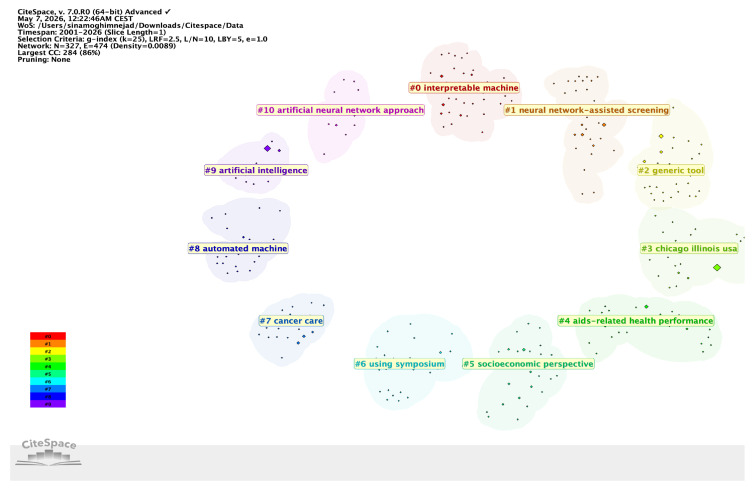
Cluster analysis of keywords in AI and healthcare policy research. Processed using CiteSpace.

The analysis identified 11 principal clusters: cluster #0, interpretable machine; cluster #1, neural network-assisted screening; cluster #2, generic tool; cluster #3, Chicago, Illinois, USA; cluster #4, AIDS-related health performance; cluster #5, socioeconomic perspective; cluster #6, using symposium; cluster #7, cancer care; cluster #8, automated machine; cluster #9, artificial intelligence; and cluster #10, artificial neural network approach. These clusters suggest that the field includes several major thematic directions, including interpretable and explainable machine learning, neural network-based screening, AI-supported tools and decision-making, public health and health performance assessment, socioeconomic and equity-related perspectives, cancer care, automated machine learning approaches, and artificial neural network methods. Some labels, such as “Chicago, Illinois, USA” and “using symposium,” appear to reflect statistically representative terms extracted by CiteSpace rather than clearly defined conceptual categories. Therefore, these clusters were interpreted cautiously in relation to their connected publications and neighboring thematic areas (Figure 16).

**Figure 17:**
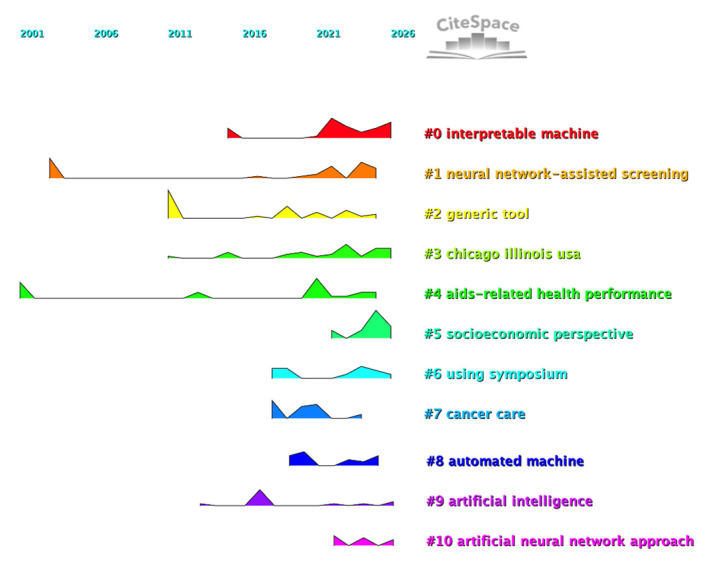
Time trend analysis of clusters in AI and healthcare policy research. Processed using CiteSpace.

The developmental trajectory of these clusters is shown in the CiteSpace timeline visualization (Figure 17). Earlier research activity was mainly associated with themes such as AIDS-related health performance, neural network-assisted screening, and generic AI-related tools, suggesting that these areas contributed to the early conceptual development of the field. In more recent years, greater attention has been noted around clusters related to interpretable machine learning, socioeconomic perspectives, artificial intelligence, automated machine learning approaches, cancer care, and artificial neural network methods. These patterns suggest that the field has gradually expanded from earlier applications of AI and computational tools toward more diverse themes involving explainability, equity, clinical implementation, and advanced machine learning approaches. Because the cluster labels were generated automatically, the temporal interpretation was limited to broad thematic patterns rather than definitive topic boundaries.

## 5. Discussion

This bibliometric analysis maps the global research landscape at the intersection of artificial intelligence and health care policy, revealing a field undergoing rapid expansion and progressively shifting from technology-centered inquiry toward governance-oriented scholarship. This study interprets these findings in the context of substantive knowledge gaps, policy implications, and emerging challenges that will shape the responsible integration of AI into health systems worldwide.

Within the examined dataset, publication activity was relatively sparse during the initial period, increased consistently from 2017 onward, and surged substantially after 2020, culminating in the highest annual output in 2025. The strong growth trajectory observed in this study is consistent with earlier bibliometric analyses showing sustained expansion of AI research in health care and medicine over the past two decades [10,17]. The present analysis extends this literature by specifically focusing on the policy dimension of AI in health care, an area that has received increasing but still comparatively limited systematic bibliometric attention.

The sharp increase in publication activity after 2020 likely reflects the convergence of technological acceleration, pandemic-driven public health needs, and increasing concern regarding the governance of AI-enabled health systems [4,37].

Prior studies have demonstrated that AI-related health research has evolved from relatively narrow technical applications toward a broader interdisciplinary field encompassing clinical medicine, public health, informatics, and health systems [10,11,13,18].

The literature has focused largely on machine learning, artificial intelligence, COVID-19, health policy, risk assessment, predictive analytics, public health, and disease-focused applications. More recent work, however, has increasingly addressed emerging areas such as large language models, interpretable machine learning, feature selection, and socioeconomic dimensions. During the pandemic, machine learning and predictive modeling were increasingly investigated for epidemiological forecasting, containment strategies, and crisis response, thereby strengthening the connection between AI research and health policy priorities [31,38,39].

The prominence of COVID-19 among the most frequent keywords supports the interpretation that the pandemic served as a major catalyst for research linking AI to disease surveillance, forecasting, resource allocation, and public health decision-making [38,40,41].

Critically, the pandemic demonstrated both the promise and limitations of AI in public health: although machine learning models were rapidly deployed for outbreak prediction and containment planning, many lacked external validation, used nonrepresentative data, and were developed without adequate attention to equity or implementation feasibility [42,43,44]. This experience underscores that publication growth alone does not equate to policy readiness and that the field must now prioritize translational rigor over volume.

The geographic distribution of publications indicates that the field is both global and unevenly concentrated. The United States emerged as the most productive country in terms of publication output, while China, England, Canada, and India were among the other leading contributors. At the same time, the present findings reveal meaningful contributions from countries outside the traditional Western research core, including India, Türkiye, Saudi Arabia, South Korea, and Iran.

In terms of centrality, the United States ranked highest, followed by England and Australia, which ranked second and third, respectively. This pattern highlights their significant intermediary roles in connecting countries within international scientific collaboration networks.

These patterns may reflect differences in research infrastructure, funding capacity, digital health ecosystems, and opportunities for multidisciplinary collaboration linking medicine, data science, and public policy. This diversification is important because the policy implications of AI in health care vary substantially across regulatory settings, health system structures, disease burdens, and data environments [9,45].

The governance challenges of AI in health care vary substantially across regulatory settings, health system structures, disease burdens, and data environments [46,47]. AI tools developed and validated predominantly in high-income settings may not perform equitably when deployed in contexts with different population demographics, disease profiles, or infrastructure constraints [1,48]. Recent evidence highlights that low- and middle-income countries face a distinct constellation of barriers to AI adoption, including unreliable internet connectivity, limited digital infrastructure, fragmented data systems, constrained regulatory capacity, workforce shortages, low AI literacy, and uncertain financing for long-term maintenance [44,46,47,48,49]. A scoping review of AI in surgical care in low- and middle-income countries found that very few studies progressed beyond development to actual deployment, underscoring the gap between algorithmic innovation and real-world clinical integration [50]. These findings reinforce calls for more equitable international collaboration, particularly in research addressing governance, access, health equity, and context-sensitive AI implementation [47,48,50].

The institutional analysis highlights the role of highly connected academic and public health centers, particularly Harvard-affiliated institutions, in shaping the development of this field. Harvard University’s high institutional centrality suggests an important bridging role connecting methodological, clinical, and policy-oriented research communities. However, the generally low institutional centrality values across the network indicate that institutional collaboration structures in this field remain relatively fragmented, consistent with an emerging and evolving domain.

In bibliometric network analysis, higher betweenness centrality is generally interpreted as evidence that a node occupies a strategically important position linking otherwise separate research communities.

Therefore, the institutional centrality findings suggest that certain universities may contribute not only through publication volume but also through their capacity to connect methodological, clinical, and policy-oriented research communities.

The journal-level findings underscore the distinctly interdisciplinary nature of AI and health care policy research. Publications were distributed across public health, nursing, medical informatics, multidisciplinary science, and general health journals. The co-citation prominence of journals such as PLOS ONE, The Lancet, JAMA, the New England Journal of Medicine, and Nature indicates that the intellectual foundation of the field draws simultaneously from technical innovation, clinical evidence, and broader health policy debates. This interdisciplinary dispersion is conceptually expected because AI policy research is not solely concerned with algorithmic performance but also with implementation, workforce implications, ethical governance, health systems, and public-sector decision-making.

The keyword and trend analyses suggest that the field is moving toward a more mature and policy-sensitive phase. Early thematic emphasis on information systems, big data, disease, and general modeling has expanded to include large language models, interpretable machine learning, feature selection, and care-related applications.

The rise of “large language models” as a recent keyword trend reflects the rapid emergence of generative AI as a transformative force in health care. These systems are increasingly being evaluated for clinical documentation, patient communication, medical education, triage, workflow support, and research assistance [7,51].

However, the literature also emphasizes substantial concerns regarding hallucinations, bias, lack of external validation, privacy, and the need for regulatory oversight [6,7].

The FDA has acknowledged that generative and agentic AI tools challenge traditional intended-use frameworks for device regulation, as these systems can perform a broad range of tasks and may therefore more closely resemble health care professionals than discrete medical devices [52].

Thus, the emergence of LLM-related topics within the bibliometric structure of this field should be interpreted not only as technological enthusiasm but also as a sign of expanding concern with governance, accountability, and evidence standards [6,53]. The rapid pace of LLM development and deployment creates an urgent need for stronger validation standards, lifecycle monitoring, and regulatory oversight tailored to the distinctive risks of generative AI in medicine and public health.

The appearance of interpretable machine learning and related themes is equally important from a policy perspective. Explainability, transparency, and interpretability are increasingly viewed as essential to clinician trust, human oversight, and responsible decision-making in high-stakes health environments [54,55].

The prominence of interpretability-related themes in the present dataset therefore aligns with broader scholarly concern that health AI systems should not only be accurate but also auditable, accountable, and socially legitimate [53,54].

This is especially relevant in public health and policy contexts, where AI outputs may influence resource allocation, population risk stratification, and regulatory or administrative decisions that affect large groups of individuals [4,5,56].

The WHO 2024 guidance and the AMIA principles both emphasize transparency as a foundational requirement for health AI governance [49,52]. However, the present bibliometric findings suggest that although interpretability is gaining traction as a research theme, the field has not yet established consensus on what level of explainability is required for different clinical and policy applications or how explainability requirements should be operationalized in regulatory standards.

The cluster analysis further reinforces the interpretation that AI and health care policy research is broadening from application-centered studies toward more diverse conceptual and governance-oriented domains. Clusters related to interpretable machine learning, socioeconomic perspectives, cancer care, neural network-assisted screening, automated machine approaches, and artificial neural network methods indicate that the field integrates technical innovation with clinical, public health, and societal concerns. The presence of clusters reflecting socioeconomic and interpretability themes is particularly significant because health AI policy increasingly focuses on fairness, inclusiveness, bias reduction, and the prevention of technology-driven inequities [53,57].

This suggests that the field is gradually shifting from the narrow question of whether AI can be applied in health care toward the more complex question of how AI should be developed, governed, validated, and deployed in ways that support equitable and trustworthy health systems [5,53].

These findings have several implications for future research and policy. First, researchers should more clearly distinguish among AI in clinical practice, AI in public health operations, and AI in health policy formulation, as these domains involve different evidence standards, ethical risks, and implementation challenges [4,56,58].

Second, the rapid rise in generative AI and large language models indicates an urgent need for stronger methodological standards, real-world validation, and post-deployment monitoring frameworks [6,7].

Third, policymakers should move beyond high-level ethical principles toward operational governance mechanisms, including data stewardship, transparency requirements, bias audits, accountability structures, and lifecycle oversight [5,53].

Finally, greater international collaboration is needed to ensure that AI governance frameworks are not shaped exclusively by a limited set of countries, institutions, or health system models [9,45].

Overall, this bibliometric analysis shows that the intersection of AI and health care policy has emerged as a rapidly developing interdisciplinary field with increasing relevance to clinical governance, public health strategy, and health system transformation. The research landscape is no longer confined to technological development alone but increasingly incorporates questions of ethics, equity, evaluation, transparency, and implementation [4,53].

As AI technologies become more deeply embedded in health systems, bibliometric mapping can help clarify where scholarship is concentrated, which themes are emerging, and where critical policy-relevant gaps remain [19,59].

## 6. Limitations

This study has several limitations that should be considered when interpreting the findings. First, the bibliometric dataset was derived exclusively from the Web of Science Core Collection. Although this database is widely used in bibliometric research, relevant publications indexed only in Scopus, PubMed, Embase, IEEE Xplore, Google Scholar, or other multidisciplinary and technical databases may not have been captured. Because database coverage varies across disciplines, journals, countries, and publication types, reliance on a single database may have influenced the observed distribution of journals, countries, institutions, collaboration networks, and research themes.

Second, the analysis was restricted to English-language peer-reviewed original articles and review articles. Although this approach improved consistency for text mining and bibliometric mapping, it may have excluded relevant studies published in other languages or in region-specific journals. This limitation is particularly important in a field such as AI and healthcare policy, where national regulatory frameworks, local implementation experiences, public-sector strategies, and policy discussions may be reported outside mainstream English-language journals.

Third, grey literature, policy documents, government reports, institutional guidelines, conference papers, editorials, letters, preprints, meeting abstracts, early access publications, and other non-article document types were excluded. Although this decision strengthened the consistency of the dataset and focused the analysis on peer-reviewed journal literature, it may have omitted important policy-relevant documents and rapidly emerging research. This is especially relevant for artificial intelligence, where conference proceedings and preprints often play a substantial role in the early dissemination of technical and policy-related developments. Therefore, the present findings may better represent the mature peer-reviewed literature than the full scope of fast-moving AI policy scholarship.

Fourth, the results may be affected by indexing bias. Bibliometric findings depend on how databases classify, index, and update records. Differences in journal coverage, citation indexing, author keywords, institutional affiliations, and subject categories may influence publication counts, citation patterns, co-citation structures, and thematic maps. Consequently, the results should be interpreted as a structured representation of the literature captured by the selected database and search strategy rather than as an exhaustive account of all research related to AI and healthcare policy.

Fifth, citation-based indicators should be interpreted cautiously. Citation counts are influenced by publication age, database coverage, journal visibility, field size, self-citation, and discipline-specific citation behavior. Older publications generally have more time to accumulate citations, which may favor foundational studies over newer but potentially influential work. Therefore, rankings of highly cited articles, authors, journals, or institutions should be understood as indicators of bibliometric visibility and scholarly influence rather than direct measures of scientific quality, clinical relevance, or policy impact.

Sixth, the interpretation of thematic clusters and keyword networks depends on the quality and consistency of indexing terms, author keywords, and software-generated labels. Cluster names generated through automated bibliometric procedures may not fully capture the conceptual complexity of the underlying literature and should therefore be interpreted as heuristic indicators rather than fixed categories. In particular, some CiteSpace-generated labels, such as “generic tool,” “using symposium,” or “Chicago, Illinois, USA,” may reflect statistically representative terms, geographic terms, or citation-context effects rather than clearly defined substantive research domains. For this reason, cluster interpretations were made cautiously and supported by the surrounding keywords, connected nodes, and broader thematic context.

Seventh, author, institutional, and country-level analyses depend on the accuracy and consistency of affiliation metadata and name disambiguation. Variations in author names, institutional names, abbreviations, organizational structures, mergers, and the coexistence of umbrella universities with affiliated medical schools may introduce minor distortions in productivity, collaboration, and centrality analyses. Although such issues are common in bibliometric research, they remain important when interpreting rankings of leading authors, institutions, countries, and collaboration hubs.

Eighth, the search strategy was based on predefined terms related to artificial intelligence and healthcare policy. As with any bibliometric query, relevant studies using adjacent concepts such as governance, regulation, reimbursement, public administration, digital health strategy, implementation science, health system transformation, or health technology assessment may not have been retrieved if they did not include the selected search terms. Accordingly, the final dataset should be viewed as a structured representation of the AI–healthcare policy literature defined by the applied search strategy rather than an exhaustive inventory of every related publication.

Ninth, the 2026 publication data should be interpreted with caution. The database search was conducted on 3 May 2026; therefore, the number of publications reported for 2026 reflects partial-year data. The apparent decline in annual output in 2026 may therefore be due to incomplete year coverage and database indexing delays rather than a true reduction in research activity.

Tenth, institutional centrality values should also be interpreted cautiously. In this study, centrality scores for institutions were generally low, suggesting that institutional collaboration networks in the field of AI and healthcare policy remain relatively fragmented. This may reflect the emerging and evolving nature of the field, in which strong institutional bridging structures have not yet fully developed. Therefore, institutional centrality should not be interpreted as evidence of dominant institutional influence but rather as an indicator of relative positioning within the available collaboration network. In addition, some institutional centrality values were very small and appeared as 0.00 after rounding because the original values were between 0 and 0.01; therefore, rankings based on very low centrality values should be interpreted carefully.

Finally, bibliometric analysis is designed to map research production, collaboration patterns, citation influence, and thematic development, but it does not directly assess methodological quality, clinical validity, policy effectiveness, implementation success, or real-world health system impact. Therefore, the present findings should be complemented by systematic reviews, scoping reviews, qualitative policy analyses, and implementation studies that evaluate the substantive quality, practical relevance, and policy implications of the identified literature.

## 7. Conclusions

This bibliometric analysis demonstrates that research on AI and health care policy has grown rapidly and become increasingly interdisciplinary, internationally distributed, and thematically diverse. The field is currently shaped by a combination of machine learning applications, public health priorities, predictive modeling, COVID-19-related research, interpretability concerns, and the rapid emergence of generative AI and large language models. The findings suggest that the literature is moving beyond a narrow focus on technological capability toward broader engagement with governance, equity, accountability, transparency, and implementation. This shift is particularly relevant to policymakers and health system leaders, as the responsible integration of AI into health care requires robust evaluation standards, ethical oversight, and context-sensitive governance frameworks.

By identifying influential contributors, leading institutions, core journals, major knowledge structures, and emerging research themes, this study provides a valuable evidence base for future scholarship and policy development in AI and health care policy. Future research should build on these findings by examining the quality, equity implications, regulatory relevance, and real-world policy impact of AI applications across diverse health systems.

## Figures and Tables

**Table 1:** Top 10 countries and institutions by number of publications. Processed using VOSviewer.

Country	Institution
USA	Harvard Medical School
China	Harvard University
England	Harvard T.H. Chan School of Public Health
Canada	University of Toronto
India	Duke University
Australia	National Taiwan University
Türkiye	National University of Singapore
South Korea	Boston University
Saudi Arabia	Ministry of Health
Iran	Sun Yat-sen University

**Table 2:** Top 10 countries and institutions by centrality. Processed using CiteSpace. Centrality values are reported as rounded values. Some values shown as 0.00 may represent very small nonzero values in the original CiteSpace output. Institutional centrality results should therefore be interpreted cautiously.

Country	Centrality	Institution	Centrality
USA	0.43	Harvard University	0.04
England	0.30	University of London	0.02
Australia	0.20	Harvard University Medical Affiliates	0.01
Saudi Arabia	0.12	Harvard T.H. Chan School of Public Health	0.01
China	0.10	Duke University	0.01
Italy	0.09	Boston University	0.01
Israel	0.09	Columbia University	0.01
India	0.08	Australian National University	0.01
Germany	0.08	Sun Yat-sen University	0.01
Turkey	0.07	University of California System	0.00

**Table 3:** Top authors by publication productivity, citation impact, and co-citation frequency. Processed using VOSviewer.

Number	Author with High Number of Publications	Number of Publications	Author with High Number of Citations	Number of Citations	The Most Co-Cited Authors	Number of Co-Citations
1	Noemi Kreif	4	Moustaq Karim Khan Rony	94	L. Breiman	46
2	Bruce Mellado	3	Mitun Debnath	90	S. Kumar	21
3	Moustaq Karim Khan Rony	3	Mst. Rina Parvin	90	T. Q. Chen	20
4	Luciano De Andrade	2	Md. Wahiduzzaman	90	MKK Rony	19
5	Oscar Kenji Nihei	2	Marek Laskowski	47	S. Athey	19
6	Joao Ricardo Nickening Vissoci	2	Bruce Mellado	44	S. M. Lundberg	19
7	Chih-Da Wu	2	Chao Yang	44	Y. Liu	18
8	Mitun Debnath	2	Ali Ahmadi	36	Z. Obermeyer	17
9	Mst. Rina Parvin	2	Ali Asgary	36	M.kuhn	16
10	Md. Wahiduzzaman	2	Jude Kong	36	Y.Wang	13

Note: This table presents three distinct author-level bibliometric indicators. “Number of publications” refers to the number of documents authored by each researcher within the included dataset. “Number of citations” refers to the total number of citations received by an author’s publications within the analyzed dataset. “Co-citation count” refers to the number of times an author was cited together with another author in the reference lists of the included publications. These metrics represent different dimensions of scholarly contribution and should not be directly compared with one another.

**Table 4:** Top 10 most cited references in AI and healthcare policy research. Processed using VOSviewer. Citation counts refer to the global citation counts recorded in the Web of Science Core Collection and processed using VOSviewer at the time of data retrieval. These values do not represent local citation counts within the 347-publication dataset.

Rank	Title of the Most Cited Paper	Year	Journal
1	Artificial intelligence and the future of global health [1]	2020	*The Lancet*
2	Artificial intelligence versus clinicians in disease diagnosis: systematic review [28]	2019	*JMIR Medical Informatics*
3	Artificial intelligence framework for simulating clinical decision-making: a Markov decision process approach [29]	2013	*Artificial Intelligence in Medicine*
4	The Human Behaviour-Change Project: harnessing the power of artificial intelligence and machine learning for evidence synthesis and interpretation [30]	2017	*Implementation Science*
5	Forecasting the dynamics of cumulative COVID-19 cases (confirmed, recovered and deaths) for the top 16 countries using statistical machine learning models: auto-regressive integrated moving average (ARIMA) and seasonal auto-regressive integrated moving average (SARIMA) [31]	2021	*Applied Soft Computing*
6	Prediction of myopia development among Chinese school-aged children using refraction data from electronic medical records: a retrospective, multicentre machine learning study [32]	2018	*PLOS Medicine*
7	Machine learning for hypertension prediction: a systematic review [33]	2022	*Current Hypertension Reports*
8	LoAdaBoost: loss-based AdaBoost federated machine learning with reduced computational complexity on IID and non-IID intensive care data [34]	2020	*PLOS ONE*
9	Priorities for successful use of artificial intelligence by public health organizations: a literature review [35]	2022	*BMC Public Health*
10	Artificial intelligence provided early detection of the coronavirus disease 2019 in China and will influence future urban health policy internationally [36]	2020	*AI*

## Data Availability

The original contributions presented in this study are included in the article/Supplementary Material. Further inquiries can be directed to the corresponding author.

## Supplementary Materials

The following supporting information can be downloaded at: https://www.mdpi.com/article/10.3390/healthcare14142103/s1, Table S1: Search strategy designed for Web of Science Database.

## Abbreviations

WoS	Web of Science
AI	Artificial Intelligence
