# Supplementary material for: Artificial Intelligence and Healthcare Policy: A Bibliometric Analysis of Global Research Trends
Source: Healthcare (Basel). 2026 Jul 14;14(14):2103. doi: 10.3390/healthcare14142103 (PMC13411476; doi:10.3390/healthcare14142103)
Supplement: Supplementary file 1 [file healthcare-14-02103-s001.zip › healthcare-4365489-supplementary.pdf]

Table S1: Search strategy designed for Web of Science Database

ISI Search formula	(("Artificial Intelligence" OR "Intelligence, Artificial" OR "Computer Reasoning" OR "Reasoning, Computer" OR "AI (Artificial Intelligence)" OR "Machine Intelligence" OR "Intelligence, Machine" OR "Computational Intelligence" OR "Intelligence, Computational" OR "Computer Vision Systems" OR "Computer Vision System" OR "System, Computer Vision" OR "Systems, Computer Vision" OR "Vision System, Computer" OR "Vision Systems, Computer" OR "Knowledge Acquisition (Computer)" OR "Acquisition, Knowledge (Computer)" OR "Knowledge Representation (Computer)" OR "Knowledge Representations (Computer)" OR "Representation, Knowledge (Computer)" OR "Machine Learning" OR "Neural Networks, Computer" OR "Computer Neural Network" OR "Computer Neural Networks" OR "Network, Computer Neural" OR "Networks, Computer Neural" OR "Neural Network, Computer" OR "Models, Neural Network" OR "Model, Neural Network" OR "Network Model, Neural" OR "Network Models, Neural" OR "Neural Network Model" OR "Neural Network Models" OR "Computational Neural Network" OR "Network, Computational Neural" OR "Networks, Computational Neural" OR "Neural Network, Computational" OR "Neural Networks, Computational" OR "Neural Network" OR "Neural Networks (Computer)" OR "Network, Neural (Computer)" OR "Networks, Neural (Computer)" OR "Neural Network (Computer)" OR "prediction model") AND ("Policies, Health" OR "Policy, Health" OR "Health Care Policies" OR "Care Policies, Health" OR "Health Care Policy" OR "Policies, Health Care" OR "Policy, Health Care" OR "Health Policies" OR "Healthcare Policy" OR "Healthcare Policies" OR "Policies, Healthcare" OR "Policy, Healthcare" OR "National Health Policy" OR "Health Policy, National" OR "National Health Policies" OR "Health Policy"))
